# The Coexistence of Antibodies to Neuronal Cell and Synaptic Receptor Proteins, Gangliosides and Selected Neurotropic Pathogens in Neurologic Disorders in Children

**DOI:** 10.3390/diagnostics13071274

**Published:** 2023-03-28

**Authors:** Karol Lubarski, Anna Mania, Sławomir Michalak, Krystyna Osztynowicz, Katarzyna Mazur-Melewska, Magdalena Figlerowicz

**Affiliations:** 1 Department of Infectious Diseases and Child Neurology, Poznan University of Medical Sciences, 27/33 Szpitalna St., 60-572 Poznan, Poland; 2 Department of Neurology, Division of Neurochemistry and Neuropathology, Poznan University of Medical Sciences, 49 Przybyszewskiego St., 60-355 Poznan, Poland

**Keywords:** N-methyl-D-aspartate receptor, autoimmune diseases, polyneuropathies, infectious diseases, child development disorders

## Abstract

Various primarily non-autoimmune neurological disorders occur synchronously with autoantibodies against tissues in the nervous system. We aimed to assess serum and cerebrospinal fluid (CSF) autoantibodies in children with neurologic disorders. To find new diagnostic tools, we compared the laboratory and clinical findings between the distinguished groups. Retrospectively, 508 patients were divided into six subgroups: neuroinfections, pediatric autoimmune neuropsychiatric disorders associated with streptococcal infections, neurologic autoimmune and demyelinating diseases, epilepsy, pervasive developmental disorders and other patients. We analysed serum anti-aquaporin-4, antiganglioside, neuronal antinuclear and cytoplasmic antibodies, as well as antibodies against surface neuronal and synaptic antigens in the CSF and serum. We involved available demographic and clinical data. Autoantibodies appeared in 165 (32.3%) children, with 24 showing multiple types of them. The most common were anti-neuroendothelium (anti-NET), anti-N-Methyl-D-Aspartate receptor (anti-NMDAr), anti-glial fibrillary acidic protein and anti-myelin antibodies bothering 46/463 (9.9%), 32/343 (9.4%), 27/463 (5.8%) and 27/463 (5.8%), respectively. Anti-NET and anti-NMDAr antibodies appeared more frequently in children with autoimmunity (*p* = 0.017; *p* < 0.001, respectively), increasing the autoimmune disease risk (OR = 2.18, 95% CI 1.13–13.97; OR = 3.91, 95% CI 1.86–8.22, respectively). Similar pathomechanisms appeared in diseases of different aetiology with clinical spectrums mimicking each other, so we proposed the model helping to diagnose autoimmune disease. We proved the influence of age, living place and medical history on the final diagnosis.

## 1. Introduction

Most neurologic symptoms non-specifically reflect the central nervous system (CNS) pathology but do not indicate the patient’s disease aetiology. Therefore, the diagnostic process involves various methods to discover the ailment basis. The complete assessment includes observation of CNS electrophysiologic function [1], structural neuroimaging [2] and measurement of biochemical compounds in bodily fluids [3,4]. However, only the complete diagnostics results matched against the clinical picture and the previous medical history lead to the precise final diagnosis [5].

We observe the lack of comprehensive multifactorial analyses in a paediatric population, including the actual laboratory revealings and past medical history and demographic data. We aim to analyse the presence of antibodies against infective agents and CNS antigens, radiologic revealings in computed tomography (CT) or magnetic resonance imaging (MRI) and the routine laboratory findings in neurological disorders of varied aetiology. Our goal is to compare the clinical data between patients with different neurologic diseases to reveal hitherto undiscovered dependencies. We seek to define the association between the preceding infections and the presence of specific autoantibodies to find the groups at risk of autoimmune diseases to accelerate the diagnostic process and introduce proper treatment. Assuming that autoimmune mechanisms occur in conditions of various aetiologies, we planned to create a mathematical model facilitating a diagnosis of primarily autoimmune diseases.

## 2. Materials and Methods

Our retrospective research involved 508 who underwent neurologic diagnostics in the tertiary centre between 3 January 2017 and 2 December 2019. The patients analysed in the study were admitted with neurological symptoms requiring testing for autoimmune disorders. Due to the non-specific clinical picture, to accelerate the diagnostic process, the differentiation of autoimmune and infectious agents was performed parallelly. We divided the group into six subgroups based on the final diagnoses: (1) patients with infections; (2) patients with autoimmune and demyelinating diseases; (3) patients with pervasive developmental disorders (PDD); (4) patients with epilepsy; (5) children with pediatric autoimmune neuropsychiatric disorders associated with streptococcal infections (PANDAS). According to the set diagnostic criteria [6,7,8], we diagnosed PANDAS, pediatric acute-onset neuropsychiatric syndrome (PANS) or pediatric infection-triggered autoimmune neuropsychiatric disorder (PITAND). The PANDAS subgroup was extracted as the independent group due to the number of participants and to avoid the potential bias associated with the differences between the PANDAS syndrome and other diseases. We verified the patients’ diagnoses after receiving all test results.

The sixth cohort involved patients who did not meet other inclusion criteria. We summarised the research groups’ final diagnoses in Table 1.

We included clinical, radiologic and laboratory data from patient-adjusted diagnostics. We analysed serum anti-aquaporin-4 antibodies in 44 patients (EUROIMMUN, Luebeck, Germany), serum antiganglioside antibodies in 99 patients (EUROIMMUN, Luebeck, Germany), serum antibodies against nuclear and cytoplasmic neural antigens in 463 patients (EUROIMMUN, Luebeck, Germany), (antineuronal nuclear antibody (ANNA) type 1 (anti-Hu), ANNA type 2 (anti-Ri), anti-Purkinje cell cytoplasmic antigen (PCA) type 1 (anti-Yo), amphiphysin, collapsin response mediator protein 5 (CRMP5) (anti-CV2), anti-Ma/Ta, anti-glutamic acid decarboxylase (GAD), anti-myelin-associated glycoprotein (MAG), anti-myelin, anti-neuroendothelium (NET), anti-Tr, anti-glial fibrillary acidic protein (GFAP), anti-PCA type 2 and against non-myelinated fibres), as well both serum and cerebrospinal fluid (CSF) antibodies against cell surface and synaptic proteins in respectively 343 and 119 patients (EUROIMMUN, Luebeck, Germany), (anti-α-amino-3-hydroxy-5-methyl-4-isoxazolepropionic receptor, anti-γ-aminobutyric acid receptor-B, anti-N-Methyl-D-Aspartate receptor (NMDAr), anti-1,3-dipropyl-8-phenylxanthine, anti-contactin-associated protein-like 2, anti-leucine-rich glioma-inactivated protein 1). We included results of herpes simplex virus (HSV)-1, HSV-2, enterovirus (EV), Epstein–Barr virus (EBV), cytomegalovirus (CMV), tick-borne encephalitis virus (TBEV), *Borrelia burgdorferi species* (*B. burgdorferi*), *Mycoplasma pneumoniae* (*M. pneumoniae*) antibodies detection. The difference in the number of tested patients with each method had various reasons, e.g., the clinical indications for particular panels. The CSF collection did not occur in several patients due to severe clinical state, unsuccessful lumbar puncture attempts, or the need to introduce therapy impacting the possible test results. Moreover, the opportunity to perform the detection of serum antibodies against nuclear and cytoplasmic neural antigens appeared before the other tests.

We performed the routine CSF tests with a standard laboratory analyser in patients with a lumbar puncture. Reference values (RV) were as follows: for CSF leukocyte count (CSF-L), RV ranged from 0–5 cells/uL; for CSF protein concentration (CSF-P), RV stayed within 15–45 mg/dL. We ran the PCR panel detecting the HSV-1, HSV-2, varicella-zoster virus (VZV), EBV, CMV, human herpesvirus (HHV)-6, HHV-7, B19 parvovirus, EV, adenovirus, parechovirus genetic material (Fast Track Diagnostics, Junglinster, Luxembourg). The antistreptolysin O (ASO) titre was measured on a standard laboratory analyser with an upper limit of normal below 150 IU/mL.

We obtained information about each patient’s living place from medical records and then classified it as a rural or an urban area according to administrative and urban planning criteria.

We created a statistical report using Statistica 13.3 (TIBCO Software Inc., 2017, Palo Alto, CA, USA). Data were compared using the χ^2^-test, the Mann–Whitney U or the Kruskal–Wallis test, suitably for the number of groups and data distribution. A *p*-value lower than 0.05 was considered statistically significant. In the Kruskal–Wallis test, we consequently checked the parameters with *p* < 0.05 with the post hoc tests. The correlations were measured with Spearman’s rank correlation coefficient for non-parametric data.

The patient’s and/or their legal guardian’s informed consent was waived due to the The Poznan University of Medical Sciences Bioethical Committee statement (19 January 2022) that our retrospective research, based on an anonymised database, has no features of a medical experiment.

## 3. Results

The participants ranged from 2.6 months (m/a) to 18 years of age (y/a), with an 8.9 y/a median. We confirmed significant differences between the analysed groups (*p* < 0.0001).

The research group involved 215 female (42%) and 293 male (58%) participants. We observed a significantly higher share of male participants in PANDAS (*p* = 0.0026; odds ratio (OR) = 3.68; 95% confidence interval (CI) 1.5–9.05) and PDD (*p* = 0.01; OR = 2.37; 95% CI 1.20–4.67) groups, whereas females dominated in the epileptic cohort (*p* < 0.0001; OR = 2.56; 95% CI 1.59–4.1). In other groups, differences were insignificant. The analysis of the living places revealed that children diagnosed with PDD more frequently resided in urban areas (*p* = 0.0401; OR = 1.98; 95% CI 1.02–3.84), whereas patients with infective diseases predominated in rural areas (*p* = 0.0013; OR = 2.83; 95% CI 1.47–5.47).

**Figure 1:**
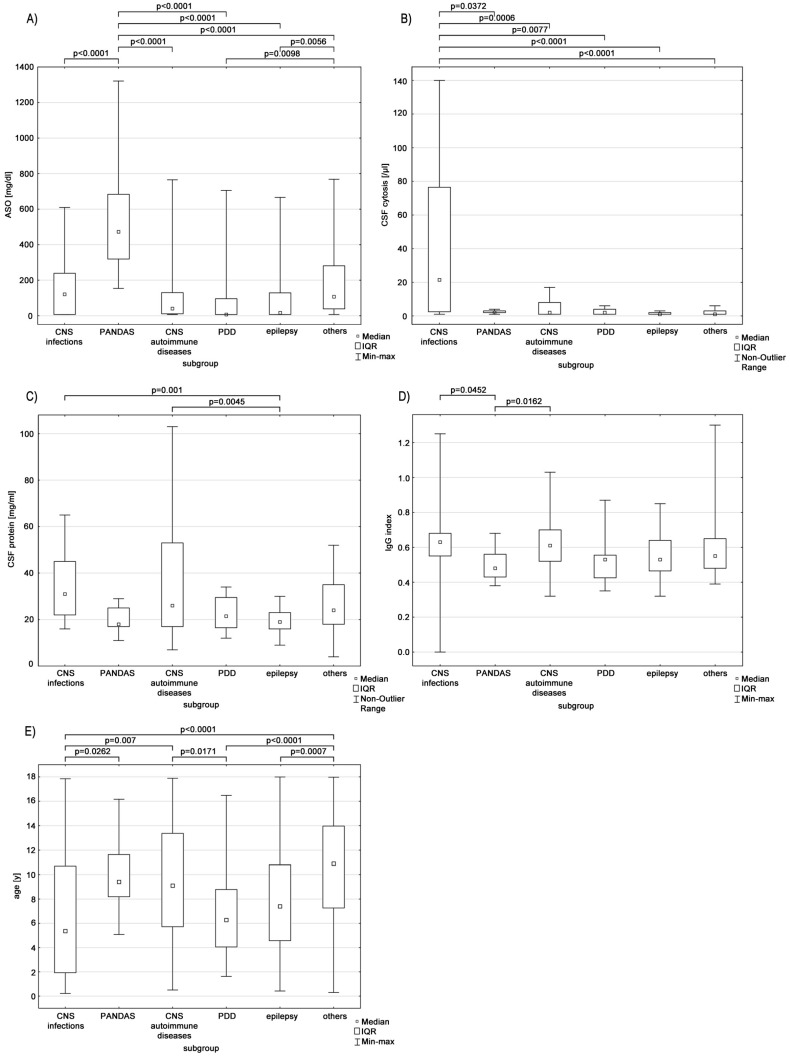
The distribution of ASO titre (**A**), CSF cytosis (**B**), CSF protein concentration (**C**), IgG index (**D**) and age (**E**). Abbreviations: ASO—antistreptolysin O, CNS—central nervous system, CSF—cerebrospinal fluid, IQR—interquartile range, PANDAS—pediatric autoimmune neuropsychiatric disorders associated with streptococcal infections, PDD—pervasive developmental disorder.

Figure 1 shows the data distributions in subdivisions.

Table 2 includes the median values and interquartile ranges (IQR).

We compared the routine CSF examinations’ results and confirmed the CSF-P difference between patients diagnosed with epilepsy and neuroinfection or autoimmune aetiology (*p* = 0.001 and *p* = 0.0045, respectively). The CSF-L was significantly higher in patients suffering from CNS infection than in any other group (*p* < 0.05). In the PANDAS cohort, the ASO titre was significantly higher (*p* < 0.01). Furthermore, patients with PDD and epilepsy had lower ASO than those in the other diseases group (*p* = 0.0098 and *p* = 0.0056, respectively).

The laboratory panel detecting serum onconeural antibodies revealed no patients with anti-Hu, anti-Ri particles and antibodies to amphiphysin or non-myelinated fibres. The most common findings included anti-NET, anti-GFAP and anti-myelin antibodies, present in, respectively, 46 (9.9%), 27 (5.8%) and 27 (5.8%) from 463 tested patients. The anti-Ma/Ta and anti-Tr antibodies appeared in one (0.2%) child; the first in the patient with multiple sclerosis with visual symptoms, and the latter in the participant with an autoimmune demyelinating process. In χ^2^ comparisons, we found anti-NET antibodies significantly more frequent in the group with autoimmune diseases (*p* = 0.0171; OR = 2.18; 95% CI 1.13–13.97). We detected the CSF anti-NMDAr antibodies in two (1.7%) of 119 tested children. However, we did not find a statistical significance (*p* = 0.1286). The mentioned antibody occurred in the serum in 32 (9.3%) of 343 screened participants. Thus, 18 of 32 positive patients had the autoimmune disease detected (*p* = 0.0002; OR = 3.91; 95% CI 1.86–8.22). We found anti-aquaporin-4 antibodies in a single patient with a non-specific visual disorder. The immunoglobulin (Ig)M and IgG against gangliosides appeared in 8 (8.1%) and 10 (10.1%), respectively, of 99 tested patients.

In our research, 261 patients underwent PCR detection of neurotrophic pathogens’ genetic material in CSF. We found nucleic acids of HHV-7 in five (1.9%), EBV in four (1.5%), EV, adenovirus and B19 parvovirus in two (0.8%) children and HSV-1, HSV-2 and HHV-6 in one (0.4%) patient. However, only the higher EV deoxyribonucleic acid frequency in the infective group showed statistical significance in the χ^2^-test (*p* = 0.0175). Moreover, in serological results, we revealed positive anti-HSV-1 IgM in 8 (3.5%) and IgG in 103 (44.8%) patients and anti-HSV-2 antibodies in IgM and IgG classes detectable in 1 (0.4%) and 8 (3.5%) cases, respectively. Anti-HSV-1 IgM antibodies occurred more frequently in the infective group than among the other participants (*p* = 0.0156). The 67 (34%) patients had anti-EV IgG antibodies, whereas only 1 (1%) were in the IgM class. In our research, anti-viral capsid antigen (VCA) EBV IgG was present in 222 (60.5%), IgM in 33 (9%) and EBV nuclear antigen (EBNA) IgG in 194 (55.7%) tested participants. Anti-CMV IgG and IgM antibodies were detected in 159 (42.2%) and 15 (4%) patients. The first mentioned was statistically less common within the infective patients’ subgroup (*p* = 0.001), whereas the latter predominated in autoimmune aetiology (*p* = 0.0009). We observed that patients with anti-CMV IgM developed 5.39 (95% CI 1.8–16.16) times more frequently an autoimmune disorder. Both IgM and IgG against HHV-6 were present in one patient. Anti-TBEV IgG antibodies appeared in four (3.9%) patients; no one was positive in the IgM class. We tested anti-*M. pneumoniae* IgG, IgM and IgA; they were detectable in 74 (20.2%), 17 (4.6%) and 10 (2.8%) children, respectively. We also found anti-*B. burgdorferi* IgG in 14 (3.7%) and IgM in 30 (7.9%) children. The anti-*B. burgdorferi* IgM was statistically more frequent in infective patients (*p* = 0.0004). All patients with neuroborreliosis had anti-*B. burgdorferi* IgM antibodies.

According to available data, 148 (33.7%) patients presented radiologic abnormalities in the CT or the MRI. The lesions appeared more often in the autoimmune group (*p* = 0.0019). The 152 (37.5%) patients who underwent EEG showed electrophysiologic disturbances, with a predominance of children diagnosed with epilepsy (*p* < 0.0001).

The ASO titre exceeded the RV in 124 out of 331 (37.5%) tested children. We confirmed the increased concentration in patients with PANDAS (*p* < 0.0001) and lower values in children with autoimmune disorders (*p* = 0.0015), PDD (*p* = 0.024) and epilepsy (*p* = 0.027). We summarised the sample sizes in the subgroups in Table 3 and Table 4.

In acquired data, the CSF-P correlated with CSF-L (*p* < 0.0001, R = 0.3969). Moreover, both parameters were associated with IgG index values (*p* = 0.0407, R = 0.1397 and *p* < 0.0001, R = 0.295, respectively). We revealed that patient age was linked with ASO titre and the CSF protein concentration (*p* < 0.0001, R = 0.443 and *p* < 0.0001, R = 0.2306, respectively).

**Figure 2:**
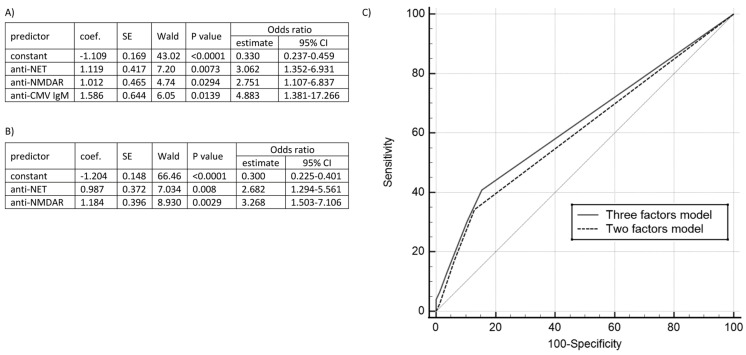
The logistic regression models. (**A**) The three-factor model characteristics (PCC = 70.71%; SP = 84.66%; SE = 40.79%). (**B**) The two-factor model characteristics (PCC = 71.97%; SP = 86.73%; SE = 34.09%). (**C**) The models’ comparison. Abbreviations: CMV—cytomegalovirus, NET—neuroendothelium, NMDAr—N-methyl-D-aspartate receptor, PCC—a percentage of consonants correct; SE—sensitivity; SP—specificity.

We constructed two logistic regression models based on statistically significant parameters: serum anti-CMV, anti-NMDAr and anti-NET positivity. The first involved all (*p* = 0.0003, R2 = 0.0765; area under the receiver operating characteristic curve (AUROC) = 0.631 ± 0.0406), and the second autoantibodies only (*p* = 0.0003, R2 = 0.0506; AUROC = 0.606 ± 0.0374); we did not notice the difference between the models (*p* = 0.348). Figure 2 depicts the complete characteristics.

## 4. Discussion

The complexity of neurologic diagnostics arises from a broad spectrum of possible underlying problems. Our research compared in subdivisions the clinical information and identified the significant differences in the CSF and the serum biochemical compounds. We confirmed the relevance of past medical history, age and living place in disease epidemiology.

### 4.1. Infectious Agents

Current national reports consider *Streptococcus pneumoniae* (*S. pneumoniae*), *Neisseria meningitidis* (*N. meningitidis*) and enteroviruses as the most common aetiologies of meningitis, and HSV-1, TBEV of encephalitis [9]. In our population, infections included VZV (11.9%), *B. burgdorferi* (9.5%) and HSV-1 (7.1%), but in 16/42 cases, the pathogenesis remained unrevealed.

In 1998, Swedo described the 50 PANDAS cases in two incidence peaks: the first between 8–12 y/o with male predominance and the second in adolescence with female predominance [7]. Our research, with a median age of 9.4 y/o with 82.4% boys’ prevalence, confirms that observation.

*M. pneumoniae*, *B. burgdorferi* and *Herpesviridae* may trigger unspecific reactions called PITAND with involuntary movements and obsessive–compulsive symptoms. Typically, the onset is abrupt, the course dynamic and the treatment complicated [6,10]. The antimicrobial antibodies often coexist, bringing the hypothesis about their co-efficiency in PITAND pathogenesis [5]. We confirmed the association between CMV infection and autoimmune reactions.

*M. pneumoniae* triggers acute neuroinfection and autoimmune reactions, causing 5–10% of children’s encephalitides [11]. We detected IgM and IgA antibodies in 4.6% and 2.8% of patients; however, the incidence did not differ between infective and autoimmune subgroups. Late neurological complications would appear in 0.1% of cases with up to 47.5% postencephalitic epilepsy if seizures accompanied the acute phase [12,13].

*B. burgdorferi* infection has an acute and chronic course but also causes developmental abnormalities due to impaired regional blood circulation damaging the cerebral white matter. Patients with borreliosis and PDD share similar temporal lobe changes: smaller cortex, hippocampus and amygdala [14,15]. The literature also suggests increased autism and tic disorder rate [16]. In our data, we observed the single anti-*B. burgdorferi*-positive PDD patient, but the share was higher in the subgroup involving PITAND, which only partially confirms the previous observations. Bransfield et al. also suspect a higher incidence of PDD in children born from borreliosis-interfered pregnancies [16].

EBV triggers myelitis, radiculopathy, meningitis and encephalitis involving Alice in Wonderland syndrome. Neurologic sequelae rates vary from 0.4% to 37.3% [17]. Valayi et al. evaluated CMV and EBV antibodies in patients with autism. The titres of IgG and IgM antibodies against CMV did not differ from the control group, whereas anti-EBV IgM increased in autistic patients [18]. Our research linked anti-CMV IgM positivity and the autoimmune disease presence, but neither EBV nor CMV correlated with PDD. The hypothesis involves the role of viral contagion in early pregnancy in autism pathogenesis, but the correlation requires further investigation [19].

HSV causes encephalitis with a 20% mortality rate and remote sequelae risk estimated at 50%, including neurological and psychomotor disturbances [20]. The association between herpesviruses and the following neurologic autoimmune reactions or epileptic episodes remains unclear. Nevertheless, while being the second most frequently reported encephalitis pathogen, HSV-1 tends to trigger autoantibody production [9,21].

### 4.2. ANA and ANCA

The analyses of antinuclear antibodies (ANA) and antineutrophil cytoplasmic antibodies (ANCA) did not reveal significant differences in our subgroups. In previous reports, autoantibodies appeared in bacterial and viral infections. Even lasting for a prolonged period, the transient ANA positivity does not equal autoimmune disease [22]. Moreover, the Th2 lymphocyte predomination in autistic patients resulted in 25% ANA positivity compared to 4% in a healthy population [23].

### 4.3. Antiganglioside Antibodies

Physiologically gangliosides spread on neurons’ surfaces enabling signal transduction and modulation. Antiganglioside antibodies occur in polyneuropathies as a significant demyelination cause [24]. The expression of specific antibodies may characterise the neuropathy type, either primary or paraneoplastic [25,26]. Our results showing the highest positivity in the autoimmune subdivision are consistent with previous reports. The anti-GM1 antibody pathogenetic role is also suspected in idiopathic epilepsy [24].

### 4.4. Antineural Antibodies

Neuroendothelial antigens occur in the entire nervous system [27]. Anti-NET antibodies appear in 1/3 of positive patients with epileptic seizures, whereas cerebellar or upper and lower motor involvement occurs in 12%, 27% and 22%, respectively [28]. The antibodies’ activity may injure the blood–brain barrier (BBB), increasing permeability and priming autoimmunity [29]. Although we noticed the significant prevalence only in the autoimmune cohort, all subgroups included positive patients. The anti-NET immunoglobulins appear in subcortical leukoencephalopathy and ataxia, including the inborn or the gluten-dependent [30]. The induction mechanism remains unknown. The hypotheses include chronic infectious diseases, e.g., tuberculosis, vasa nervosum vasculitis and underlying rheumatic disorders [29].

The GFAP is an astroglial tissue marker. In our research, 9.9% of patients were anti-GFAP-positive but without a predominance in any subgroup. Typically, the antibodies accompany autoimmune demyelinating diseases, affecting the cortical, subcortical or spinal areas and resulting in cognitive and sensorimotor disturbances or seizures [31]. In addition to spontaneous induction, an infection may also trigger their production [32]. In 2018, Li and colleagues described the first autoimmune GFAP-mediated astrocytopathy after HSV-1 sickness [32]. Nevertheless, the role of serum anti-GFAP is limited. To confirm the anti-GFAP-associated disorder, the detection of antibodies in the intrathecal compartment is needed. Serum-only-positive anti-GFAP patients are more likely to suffer from non-inflammatory neurological diseases than neuroinflammation [33].

Anti-myelin antibodies appear in neurological autoimmune diseases, glaucoma, neoplasms and diabetes mellitus type 1 (DM1). Clinically, these antibodies cause cerebellar and motor neuron disorders due to primary degeneration or secondary response to damage [28]. Both anti-myelin oligodendrocyte glycoprotein with anti-major basic protein belongs to early and late multiple sclerosis (MS) predictors. The first mentioned appears in neuromyelitis optica cases, acute disseminated encephalomyelitis (ADEM) and transverse myelitis pathogeneses in, respectively, 2.1–5.25%, 40–68% and 20% [34]. Persistent seropositivity connects with 3–5 times more significant relapse risk [35]. The anti-MAG is characteristic of IgM monoclonal gammopathy of uncertain significance polyneuropathy, which shows over 50% seropositivity; however, anti-MAG appears in 5.6% of patients without monoclonal gammopathy [36]. In our research, 15 children produced anti-MAG antibodies, none with gammopathy; the highest percentage appeared in the PANDAS group.

### 4.5. Onconeural Antibodies

Anti-PCA2 antibodies accompany headaches, cognitive impairment, peripheral paraesthesia and spastic paresis. The antibody may appear in paraneoplastic syndromes connected with lymphomas, neuroblastoma and neuroendocrine tumours [37,38]. Our patients suffered from autoimmune neuroinflammation, epilepsy and PDD.

Anti-Yo antibodies may trigger neoplastic syndromes, mainly connected with breast carcinoma and ovarian tumours, less frequently with Hodgkin lymphoma [37,39]. However, none of our six positive patients was diagnosed with malignancy. The particles were also described in non-oncologic children with attention deficit hyperactive disorder [40].

Anti-GAD antibodies typically link with DM1, but increased titre may trigger neurological symptoms [41]. CNS involvement is rare but hardly responds to immunotherapy and antiepileptic drugs. The disease connects with focal seizures, temporal lobe epilepsy and cognitive or memory impairment [21]. Interestingly, 40% of our anti-GAD-positive children were diagnosed with epilepsy.

### 4.6. Anti-NMDAr Autoantibodies

Autoimmune disorders with antibodies against cell surface and synaptic proteins require comprehensive diagnostics, including detecting triggering factors in serum and CSF, EEG and CNS imaging. Although the guidelines suggest the simultaneous analysis of serum and CSF in any autoimmune disorder suspicion [42], the latter material remains more diagnostically valuable [43]. Our research group involved two (1.7%) patients with anti-NMDAr detectable in CSF and 32 (9.4%) in peripheral blood. One child was diagnosed with a brain stem tumour.

Anti-NMDAr is associated with the most common autoimmune encephalitis, but antibodies also appear in paraneoplastic and post-infective syndromes [10]. In the general population, 58% of cases associate with ovarian teratoma; in male patients, the neoplasm occurs in 5% [39,44]. In the paediatric population, post-infective syndromes dominate. HSV appears as the best-recognised triggering factor, causing chorea, ballism, ataxia and psychiatric disorders in 70% of patients preceded by flu-like symptoms [21,37,42]. The animal model proved that anti-NMDAR antibodies might cross the placenta and affect the foetus, causing neuropsychiatric disturbances [45].

### 4.7. Radiologic Findings

Routine neurologic diagnostics require radiological imaging. Neuroinfection may manifest with characteristic lesions or mimic autoimmune abnormalities [2]. Endres et al. estimated that almost 86% of patients with antibodies against intracellular antigens reveal lesions in MRI [46]. In our population, less than half of the patients with autoimmune disorders and only a quarter with infection had any MRI/CT abnormality. The available reports describe characteristic patterns for specific autoantibodies, but the overlapping radiologic findings force performing other specialistic laboratory tests in differentiation [2,32,37].

### 4.8. Electroencephalography

Epileptic seizures result from cerebral electrophysiologic disturbances originating from structural abnormalities, inherited predispositions, metabolic disorders or infection. Essentially, paroxysmal convulsions during the neuroinfection do not equate to the infective aetiology of epilepsy [47]. Our data showed a statistical increase in female patients in the subgroup with epilepsy, confirming the predisposition widely described in the literature [48]. The antiphospholipid antibodies and ANA may reflect the autoimmune epilepsy basis in children [49].

### 4.9. Place of Living

Limited data confirm the correlation between urbanisation and higher PDD or autism prevalence. The incidence rate ratios (IRRs) in the urban areas compared to rural were assessed at 1.81 and 1.45, respectively [50]. Autism prevalence in urban areas was also described in Taiwan [51]. The reason remains unknown; however, burdened families’ migration is hypothesised.

The information about the city’s higher neuroinfection incidence comes from highly urbanised countries and correlates with population density [52]. Contrary to this, we observed a protective influence on urban areas. The possible explanation includes more probable contact with pathogens that may be associated with animal husbandry in rural zones.

### 4.10. Logistic Regression Models

We created the tool to estimate the autoimmunity risk based on our statistically proven factors. Expanding the two-factor model with anti-CMV IgM increases the sensitivity by 6%, with specificity decreasing by 2%. Both models have comparable, relatively high specificity (84.7% and 86.7%, respectively), enabling the patients’ preliminary selection for further diagnostics. The complete characteristics are shown in Figure 2.

## 5. Conclusions

The courses of disorders with different aetiologies overlap, requiring multidirectional diagnostics. Additionally, identical immunoglobulins primarily appear independently in autoimmune diseases, as well as secondarily in infections or as a part of an infection, and they may proceed with autoantibodies production or paraneoplastic disorders. On the other hand, the positivity share in the asymptomatic population is unknown. For example, anti-NET showed significant prevalence in our research in the autoimmune cohort, but anti-NET-positive patients appeared in all research subgroups. In our population, we noticed the strongest correlation of anti-CMV infection, which increases the autoimmune disorder risk over five times (95% CI 1.8–16.16). Moreover, we revealed that the place of living impacts disease incidence—the predomination of infectious diseases in rural and PDD in urban areas. The quicker diagnosis was made thanks to indicating risk factors that would lead to an introduction of adjusted treatment in the earlier phase of the disease and result in reduced sequelae risk.

A recent study reports that the prevalence of autoimmune encephalitides may be comparable to all infection-triggered cases [53]. Due to that fact, we suggest including autoantibody testing in the diagnostic process. Analogously to paraneoplastic syndromes, we see the need to simultaneously test serum and CSF due to the different specificity and sensitivity of tests for a particular autoantibody and each material [54]. Moreover, due to the overlapping clinical manifestations [55], physicians cannot discriminate doubts about the triggering autoantibody. Because of this, we see the indications for multiantigen-panel testing to reduce the risk of culprit omission.

The main limitations of our research result from incomplete clinical and laboratory data associated with the retrospective character. However, the presented data, showing the significant incidence of particular autoantibodies, motivate further research. We believe that a prospective study with a standardised diagnostic protocol would improve the strength of the conclusions and lead to valuable clinical discoveries. The co-appearance of antibodies against nuclear, cytoplasmic and cell surface neural or synaptic proteins in non-oncologic disorders in children belongs to the developing branch of research. We hold that our work is a step ahead in understanding autoimmune diseases, their risk factors and diagnostics.

## Figures and Tables

**Table 1:** Group sizes with subdivisions based on the final diagnoses.

CNS Infections (*n* = 42)		PANDAS (*n* = 34)		Autoimmune and Demyelinating Diseases (*n* = 139)		PDD (*n* = 48)		Epilepsy (*n* = 88)		Other (*n* = 157)	
diagnosis	*n*	ass. symptoms	*n*	diagnosis	*n*	diagnosis	*n*	diagnosis	*n*	diagnosis	*n*
**Neuroborreliosis**	**4**	**tics**	**27**	**Neuropathies**	**44**	**Autism**	**37**	**generalised idiopathic:**	**58**	**involuntary movements diagnostics**	**41**
**meningitis:**	**12**	**OCD**	**10**	- GBS	13	**Asperger syndrome**	**3**	- tonic-clonic	15	**headache**	**22**
- HHV-7	2	**anxiety** **disorder**	**3**	- mononeuropathies	6	**Other PDD**	**8**	- polymorphic	11	**dissociative and emotional disorders**	**21**
- HSV-1 + *N. meningitidis*	1	**psychomotor** **delay**	**4**	- CIDP	5			- absence	8	**movement disorders**	**11**
- Influenza A virus	1	**seizures**	**3**	- postinfectious neuropathies	5			- myoclonic	8	- tetraplegia	1
- unidentified agent	8	**visual** **symptoms**	**3**	- axonal neuropathy	10			- other generalised seizures	16	- dizziness, imbalance	8
**encephalitis:**	**12**			- other and undescribed	5			**focal idiopathic:**	**19**	- ataxia and gait impairment	2
- VZV	4			**CNS inflammation:**	**44**			- with generalisation	12	**neoplasms and paraneoplastic syndromes**	**9**
- HSV-1	2			- encephalitis	31			- simple focal	7	- ALL	3
- HHV-6	1			- cerebellitis	4			**symptomatic**	**11**	- spinal tumour	2
- Influenza A virus	1			- ADEM	3					- Neuroblastoma suspicion, OMS	2
- rotavirus	1			- encephalomyelitis	2					- brain stem tumour	1
- enterovirus	1			- encephalomeningitis	1					- thalamic tumour	1
- unidentified agent	2			- myelitis	1					**psychomotor delay**	**9**
**cerebellitis and ataxia:**	**5**			- acute demyelinating episode	1					**vision disorders**	**9**
- VZV	1			- Rasmussen syndrome	1					**neuromuscular or muscular disease**	**7**
- *M. pneumoniae*	1			**Optic nerve inflammation**	**7**					**CNS diagnostics in** **systemic rheumatologic diseases**	**6**
- unidentified agent	3			**PANS**	**7**					**stroke or TIA**	**5**
**meningocerebellitis:**	**2**			**PITAND**	**6**					**other**— **differential diagnosis**	**17**
- EBV	1			**MS**	**6**					- gastroenterological symptoms	4
- enterovirus	1			**Myasthenia**	**1**					- appendicitis	1
**encephalomeningitis:**	**3**			**Other:**	**24**					- MRI abnormalities	4
- *S. pneumoniae*	1			- CNS demyelination	14					- Idiopathic scoliosis	1
- unidentified agent	2			- involuntary movement disorder reactive to IVIG	7					- febrile seizures	2
**panencephalitis**	**1**			- progressive encephalopathy	1					- heat-illness	1
**Cerebral toxocariasis**	**1**			- MS diagnostics	1					- transient neurological symptoms	2
***M. pneumoniae*-induced:**	**2**			- autoimmune mediated development delay suspicion	1					- respiratory abnormalities, dyspnoea	1
- tic disorder	1									- inborn error of metabolism suspicion	1
- OMS	1										

Abbreviations: ADEM—acute disseminated encephalomyelitis, ALL—acute lymphocytic leukaemia, CANS—childhood acute neuropsychiatric symptoms, CIDP—chronic inflammatory demyelinating polyneuropathy, CNS—central nervous system, EBV—Ebstein–Barr virus, GBS—Guillain–Barré syndrome, HHV—human herpes virus, HSV—herpes simplex virus, IVIG—intravenous immunoglobulin, *M. pneumoniae*—*Mycoplasma pneumoniae*, MS—multiple sclerosis, *N. meningitidis*—*Neisseria meningitidis*, OCD—obsessive–compulsive disorder, OMS—opsoclonus myoclonus syndrome, PANDAS—pediatric autoimmune neuropsychiatric disorders associated with streptococcal infections, PANS—pediatric acute-onset neuropsychiatric syndrome, PDD—pervasive developmental disorders, PITAND—pediatric infection-triggered autoimmune neuropsychiatric disorder, *S. pneumoniae*—*Streptococcus pneumoniae*, TIA—transient ischemic attack, VZV—varicella–zoster virus.

**Table 2:** Median and IQR values of analysed parameters.

	All (*n* = 508) Median (IQR)	CNS Infections (*n* = 42) Median (IQR)	PANDAS (*n* = 34) Median (IQR)	Autoimmune and Demyelinating Diseases (*n* = 139) Median (IQR)	PDD (*n* = 48) Median (IQR)	Epilepsy (*n* = 88) Median (IQR)	Other (*n* = 157) Median (IQR)
age (y/a)	8.9 (5.4–12.7)	5.4 ^bcf^ (1.9–10.7)	9.4 ^a^ (8.2–11.6)	9.1 ^ad^ (5.7–13.4)	6.3 ^cf^ (4.1–8.8)	7.4 ^f^ (4.6–10.8)	10.9 ^ade^ (7.3–14)
CSF protein (mg/dL)	24 (17–38)	31 ^e^ (22–45)	18 (17–25)	26 ^e^ (17–53)	21.5 (16.5–29.5)	19 ^ac^ (16–23)	24 (18–35)
CSF cytosis (/µL)	2 (1–6)	22 ^bcdef^ (3–77)	2 ^a^ (2–3)	2 ^a^ (1–8)	2 ^a^ (1–4)	1 ^a^ (1–2)	1 ^a^ (1–3)
IgG index	0.57 (0.49–0.67)	0.63 ^b^ (0.55–0.68)	0.48 ^ac^ (0.43–0.56)	0.61 ^b^ (0.52–0.70)	0.53 (0.43–0.56)	0.53 (0.47–0.64)	0.55 (0.48–0.65)
ASO (IU/mL)	74 (7–268)	120.5 ^b^ (7–239)	472.5 ^acdef^ (319–684)	40 (11–130)	7 ^bf^ (7–96)	17 ^bf^ (7–129)	107 ^bde^ (39–281)

^a^—comparison with infective group *p* < 0.05; ^b^—comparison with PANDAS group *p* < 0.05; ^c^—comparison with autoimmune group *p* < 0.05; ^d^—comparison with PDD group *p* < 0.05; ^e^—comparison with epilepsy group *p* < 0.05; ^f^—comparison with group with other diseases group *p* < 0.05. Abbreviations: ASO—antistreptolysin O, CNS—central nervous system, CSF—cerebrospinal fluid, IgG—immunoglobulin G, IQR—interquartile range, PANDAS—pediatric autoimmune neuropsychiatric disorders associated with streptococcal infections, PDD—pervasive developmental disorders, y/a—years of age.

**Table 3:** Summary of demographic, radiologic and selected autoimmunity diagnostics.

		Without Division *n* (%)	Subgroups					
			CNS Infections *n* (%)	PANDAS *n* (%)	Autoimmune and Demyelinating Diseases *n* (%)	PDD *n* (%)	Epilepsy *n* (%)	Other *n* (%)
sex	female	215 (42.3)	16 (38.1)	6 (17.6)	60 (43.2)	12 (25)	54 (61.4) *	67 (42.7%)
	male	293 (57.7)	26 (61.9)	28 (82.4) *	79 (56.8)	36 (75) *	34 (38.6)	90 (57.3%)
living place	rural	208 (40.9)	27 (64.3) *	12 (35.3)	57 (41)	13 (27.1)	35 (39.8)	64 (40.8%)
	urban	300 (59.1)	15 (35.7)	22 (64.7)	82 (59)	35 (72.9) *	53 (60.2)	93 (59.2%)
IgG index ≥0.75		43/216 (19.9%)	6/27 (24)	0/17 (0) **	23/93 (24.7)	1/12 (8.3)	3/20 (15)	10/49 (20.4)
anti-MAG Ab		15/463 (3.2%)	0/38 (0)	3/32 (9.4)	5/123 (4.1)	0/45 (0)	1/85 (1.2)	6/140 (4.3)
anti-myelin Ab		27/463 (5.8%)	4/38 (10.5)	2/32 (6.3)	8/123 (6.5)	0/45 (0)	5/85 (5.9)	8/140 (5.7)
anti-NET Ab		46/463 (9.9%)	1/38 (2.6)	3/32 (9.4)	19/123 (15.4) *	4/45 (8.9)	11/85 (12.9)	8/140 (5.7) **
anti-GFAP Ab		27/463 (5.8%)	2/38 (5.3)	4/32 (12.5)	6/123 (4.9)	4/45 (8.9)	6/85 (7.1)	5/140 (3.6)
serum anti-NMDAr Ab		32/343 (9.3%)	1/32 (3.1)	1/31 (3.2)	18/95 (18.9) *	3/32 (9.4)	4/60 (6.7)	5/93 (5.4)
ANA		114/310 (36.8%)	10/24 (41.7)	10/27 (37)	36/93 (38.7)	6/29 (20.7)	18/45 (40)	34/92 (37)
MRI/CT lesions		148/439 (33.7%)	11/41 (26.8)	7/28 (25)	56/125 (44.8) *	6/38 (15.8)	28/80 (35)	40/127 (31.5)

* *p* < 0.05—more prevalent in the subgroup. ** *p* < 0.05—less prevalent in the subgroup. Abbreviations: Ab—antibody, ANA—antinuclear antibody, CNS—central nervous system, CSF—cerebrospinal fluid, CT—computed tomography, GFAP—glial fibrillary acidic protein, MAG—myelin-associated glycoprotein, MRI—magnetic resonance imaging, NET—neuroendothelium, NMDAr—N-methyl-D-aspartate receptor, PANDAS—pediatric autoimmune neuropsychiatric disorders associated with streptococcal infections, PDD—pervasive developmental disorders.

**Table 4:** Results of selected infective agents’ diagnostics in the research group with and without subdivisions.

	Without Division *n* (%)	Subgroups					
		CNS Infections *n* (%)	PANDAS *n* (%)	Autoimmune and Demyelinating Diseases *n* (%)	PDD *n* (%)	Epilepsy *n* (%)	Other *n* (%)
anti-HSV-1 IgG Ab	103/230 (44.8)	11/32 (34.4)	7/14 (50)	40/84 (47.6)	9/18 (50)	16/38 (57.1)	20/54 (37)
anti-HSV-1 IgM Ab	8/226 (3.5)	4/32 (12.5) *	0/14 (0)	3/85 (3.5)	0/14 (0)	1/28 (3.6)	0/53 (0)
anti-HSV-2 IgG Ab	8/229 (3.5)	1/32 (3.1)	1/13 (7.7)	4/84 (4.8)	0/18 (0)	0/28 (0)	2/54 (3.7)
anti-HSV-2 IgM Ab	1/224 (0.4)	0/32 (0)	0/13 (0)	1/85 (1.2)	0/14 (0)	0/28 (0)	0/52 (0)
anti-VCA EBV IgG Ab	222/367 (60.5)	18/33 (54.5)	14/24 (58.3)	69/112 (61.6)	20/37 (54.1)	37/58 (63.8)	64/103 (62.1)
anti-VCA EBV IgM Ab	33/368 (9)	3/33 (9.1)	1/24 (4.2)	13/112 (11.6)	2/37 (5.4)	5/59 (8.5)	9/103 (8.7)
EBNA IgG Ab	194/348 (55.7)	15/31 (48.4)	13/24 (54.2)	58/104 (55.8)	19/37 (51.4)	32/55 (58.2)	57/97 (58.8)
anti-CMV IgG Ab	159/377 (42.2)	5/33 (15.2) **	12/26 (46.2)	44/99 (40.4)	16/36 (44.4)	30/62 (48.4)	52/111 (46.8)
anti-CMV IgM Ab	15/377 (4)	0/33 (0)	0/25 (0)	10/108 (9.3)*	0/36 (0)	0/62 (0)	5/113 (4.4)
anti-*B. burgdorferi* IgG Ab	14/378 (3.7)	2/34 (5.9)	2/30 (6.7)	7/118 (5.9)	0/31 (0)	1/55 (1.8)	2/110 (1.8)
anti-*B. burgdorferi* IgM Ab	30/380 (7.9)	8/34 (23.5) *	0/30 (0)	11/119 (9.2)	1/31 (3.2)	2/56 (3.6)	8/110 (7.3)
anti*-M. pneumoniae* IgG Ab	74/366 (20.2)	7/36 (19.4)	8/28 (28.6)	16/110 (14.5)	2/30 (6.7)	11/62 (17.7)	30/100 (30) *
anti-*M. pneumoniae* IgM Ab	17/368 (4.6)	3/36 (8.3)	1/28 (3.6)	7/111 (6.3)	0/31 (0)	0/62 (0)	6/100 (6)
anti-*M. pneumoniae* IgA Ab	10/360 (2.8)	1/35 (2.9)	0/28 (0)	3/109 (2.8)	0/29 (0)	1/62 (1.6)	5/97 (5.2)

* *p* < 0.05—more prevalent in the subgroup. ** *p* < 0.05—less prevalent in the subgroup. Abbreviations: Ab—antibody, *B. burgdorferi*—*Borrelia burgdorferi species*, CMV—cytomegalovirus, CNS—central nervous system, EBNA—Epstein–Barr nuclear antigen, EBV—Ebstein–Barr virus, HSV—herpes simplex virus, IgA—immunoglobulin A, IgG—immunoglobulin G, IgM—immunoglobulin M, *M. pneumoniae*—*Mycoplasma pneumoniae*, PANDAS—pediatric autoimmune neuropsychiatric disorders associated with streptococcal infections, PDD—pervasive developmental disorders, VCA—viral capsid antigen.

## Data Availability

Data sharing is not applicable.
